# Incremental Prognostic Value of the Incorporation of Clinical Data Into Coronary Anatomy Data in Acute Coronary Syndromes: SYNTAX-GRACE Score

**DOI:** 10.5935/abc.20170160

**Published:** 2017-12

**Authors:** Mateus dos Santos Viana, Fernanda Lopes, Antonio Mauricio dos Santos Cerqueira Junior, Jessica Gonzalez Suerdieck, André Barcelos da Silva, Ana Clara Barcelos da Silva, Thiago Menezes Barbosa de Souza, Manuela Campelo Carvalhal, Marcia Maria Noya Rabelo, Luis Claudio Lemos Correia

**Affiliations:** 1 Escola Bahiana de Medicina e Saúde Pública, Salvador, BA - Brazil; 2 Hospital São Rafael, Fundação Monte Tabor, Salvador, BA - Brazil

**Keywords:** Acute Coronary Syndrome / prognosis, Coronary Artery Disease, Cardiac Catheterization

## Abstract

**Background:**

When performing coronary angiography in patients with acute coronary syndrome (ACS), the anatomical extent of coronary disease usually prevails in the prognostic reasoning. It has not yet been proven if clinical data should be accounted for in risk stratification together with anatomical data.

**Objective:**

To test the hypothesis that clinical data increment the prognostic value of anatomical data in patients with ACS.

**Methods:**

Patients admitted with objective criteria for ACS and who underwent angiography during hospitalization were included. Primary outcome was defined as in-hospital cardiovascular death, and the prognostic value of the SYNTAX Score (anatomical data) was compared to that of the SYNTAX-GRACE Score, which resulted from the incorporation of the GRACE Score into the SYNTAX score. The Integrated Discrimination Improvement (IDI) was calculated to evaluate the SYNTAX-GRACE Score ability to correctly reclassify information from the traditional SYNTAX model.

**Results:**

This study assessed 365 patients (mean age, 64 ± 14 years; 58% male). In-hospital cardiovascular mortality was 4.4%, and the SYNTAX Score was a predictor of that outcome with a C-statistic of 0.81 (95% CI: 0.70 - 0.92; p < 0.001). The GRACE Score was a predictor of in-hospital cardiac death independently of the SYNTAX Score (p < 0.001, logistic regression). After incorporation into the predictive model, the GRACE Score increased the discrimination capacity of the SYNTAX Score from 0.81 to 0.92 (95% CI: 0.87 - 0.96; p = 0.04).

**Conclusion:**

In patients with ACS, clinical data complement the prognostic value of coronary anatomy. Risk stratification should be based on the clinical-anatomical paradigm, rather than on angiographic data only.

## Introduction

For a patient with acute coronary syndrome (ACS) undergoing invasive stratification by use of cardiac catheterization, coronary anatomy assessment is used to guide treatment, identifying the lesion related to the clinical event, providing the necessary information to establish the best treatment strategy, such as surgical and percutaneous revascularization, in addition to providing short- and long-term prognostic information.^1^ In the decision-making process, once knowing the coronary anatomy, it is uncertain if the clinical data should influence the treatment choice.

The SYNTAX Score was initially created to assess the extent of the coronary artery disease (CAD) as well as the feasibility of the percutaneous coronary intervention in patients with stable CAD,^2^ and proved to be a good long-term prognostic marker in several CAD scenarios, such as that of patients with ACS.^3,4^

The GRACE Risk Score is widely used in daily medical practice to stratify the risk of patients with ACS, incorporates several clinical variables into its model,^5^ and has a higher ability to predict events as compared to other risk scores.^6^ However, once the coronary anatomy is known, it is not clear if the GRACE Score should be incorporated into the clinical decision-making process, or if it should be used only to define the invasiveness of the initial strategy.

The objective of this study is to test the hypothesis that clinical data, specifically represented by the GRACE Score, increment the prognostic value of the anatomical assessment provided by using the SYNTAX Score, in addition to assessing its clinical usefulness. Therefore, incremental value analysis, C-statistic discrimination and net reclassification analysis of the new predictive model were performed.

## Methods

### Population Selected

Individuals consecutively admitted to the Intensive Cardiovascular Unit of two tertiary hospitals, between August 2007 and October 2014, and diagnosed with ACS (RESCA Registry) were selected. The inclusion criterion of this registry was defined as typical chest discomfort and at rest in the previous 48 hours associated with at least one of the following characteristics: 1) positive myocardial necrosis marker, defined as troponin T ≥ 0.01 µg/L or troponin I > 0.034 µg/L, which correspond to values above the 99th percentile;^7,8^ 2) ischemic electrocardiographic changes, consisting of T-wave inversion (≥ 0.1 mV) or ST-segment deviation (≥ 0.05 mV); 3) documented CAD, defined as history of myocardial infarction or previous angiography showing coronary obstruction ≥ 50% of the luminal diameter.

For the present analysis, patients included in the registry who underwent coronary angiography during the treatment were selected. Individuals who refused to participate in the registry and those who had previously undergone myocardial revascularization surgery were excluded. The study protocol is in accordance to the Declaration of Helsinki, was approved by the Ethics Committee in Research of the institutions, and all patients provided written informed consent.

### SYNTAX and GRACE Scores

For this study, the SYNTAX Score was calculated by an experienced interventional cardiologist, blinded to the chosen treatment modality, to the clinical findings and to the primary outcome, and who assessed every coronary obstruction ≥ 50% in vessels whose diameter was ≥ 1.5 mm, following the tutorial described in a previous study.^9^ That tutorial considered several angiographic parameters, such as lesion location and number of vessels affected, presence of bifurcation or ostial lesion, total vascular occlusion, occlusion time, presence of collateral circulation, lesion extent, presence of thrombi, significant tortuosity, excessive calcification, and diffuse disease.

The GRACE Score was calculated on patient’s admission, and consisted of eight variables: five computed semi-quantitatively, that is, with a different weight for each stratum of age, systolic blood pressure, heart rate, serum creatinine and Killip class; and three computed dichotomously: ST-segment depression, elevation in myocardial necrosis marker, and cardiac arrest on admission. The final score can range from 0 to 372.^5^

In addition to collecting the clinical and angiographic variables used for calculating the scores, the occurrence of left ventricular dysfunction was assessed, defined as Simpson’s method ejection fraction (EF) < 45%, a mean value corresponding to the classification of mild ventricular dysfunction (EF between 40% and 49%), in accordance with the most recent guidelines on echocardiography.^10^

### In-hospital clinical outcome

The variable ‘primary outcome’ was defined as in-hospital cardiovascular death. Cardiovascular death was defined as sudden death or cardiovascular hospitalization followed by death.

### Data analysis

Initially, a Receiver Operating Characteristic (ROC) curve was built with the GRACE Score values to predict cardiovascular outcome. Once its accuracy was obtained in the ROC curve, the GRACE Score entered the logistic regression model with the SYNTAX Score. If the GRACE Score reached statistical significance at the 5% level (p < 0.05), the new SYNTAX-GRACE Score would be created by adding points when the GRACE Score was high. The additional points were determined by dividing the regression coefficient of the high GRACE Score by the regression coefficient of the SYNTAX Score. In the discrimination analysis, the C-statistics of the SYNTAX and SYNTAX-GRACE models were compared by using Hanley-McNeil test. The calibration of the models was described with the Hosmer-Lemeshow test. In addition, the ability of the new model (SYNTAX-GRACE) to correctly reclassify the information from the traditional SYNTAX model was assessed. This reclassification analysis used the method proposed by Pencina to calculate the *Integrated Discrimination Improvement* (IDI).^11^

The categorical variables were expressed as absolute and percentage numbers, the values of the scores were expressed as median and interquartile interval (IQI) and compared between the groups by using the nonparametric Mann-Whitney test. Statistical significance was defined as p value < 0.05. The SPSS Statistical Software (version 21.0, SPSS Inc., Chicago, Illinois, USA) and the MedCalc Software (version 12.3.0.0, Mariakerke, Belgium) were used for data analysis, and the latter for comparing between the ROC curves.

### Calculating the sample size

The sample size was calculated to offer statistical power to two predefined analyses. First, logistic regression analysis, in which the predictive value of the GRACE Score was assessed independently from the SYNTAX Score. Because that analysis requires two covariables (high GRACE and high SYNTAX), 20 outcomes would be necessary to maintain the relationship recommended of 10 outcomes per covariable.^12^ Expecting a 10% incidence of outcomes, at least 200 patients would be necessary. Second, the comparison of the C-statistic of SYNTAX *versus* SYNTAX-GRACE: adopting the assumption of the 0.95 correlation coefficient between the values of the two models to reach a statistical power of 80% (one-tailed alpha of 0.05) to detect 0.05 of C-statistic superiority (for example, 0.65 *versus* 0.70) of the more complete model (SYNTAX-GRACE), the analysis would need to include at least 192 patients.

## Results

During the study period, 822 patients were included in the RESCA registry, 370 of whom underwent coronary angiography and 5 patients had undergone previous revascularization surgery. Regarding the 365 patients assessed, their mean age was 64 ± 14 years, 58% were of the male sex, 54% had non-ST-segment elevation myocardial infarction, 27% had unstable angina, and the rest had ST-segment elevation myocardial infarction. Killip classification > I and presence of systolic dysfunction, defined as EF < 45% on the echocardiogram, were observed in 14% and 13% of the patients, respectively. Significant coronary disease with three-vessel or left main coronary artery involvement was identified in 36% of the sample.

**Figure 1:**
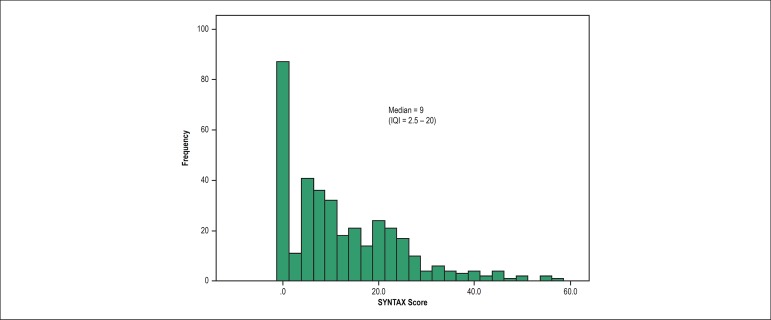
Histogram of frequency of the SYNTAX Score in the population studied

The median SYNTAX Score was 9 (IQI: 2.5 - 20; Figure 1), and the median GRACE Score was 117 (IQI: 90 - 144). Analyzing the risk tertiles predicted in the SYNTAX Study,^2^ 81.4% of the patients had a low SYNTAX Score (0 to 22), 10.1% had an intermediate SYNTAX Score (23 to 32), and only 8.5% had a high SYNTAX Score (> 33). Sixteen patients (4.4%) had in-hospital cardiovascular death. Other relevant clinical characteristics are shown in Table 1.

**Table 1:** Clinical and angiographic characteristics and occurrence of the outcome in the patients studied

Variables	N
Sample size	365
Age (years)	64 ± 14
Male sex	210 (57.7%)
Ischemia on electrocardiogram	166 (45.6%)
Unstable angina	98 (26.8%)
NSTEMI	196 (53.7%)
STEMI	71 (19.5%)
Positive troponin	232 (63.7%)
Three-vessel or LMC	122 (36.6%)
GRACE Score*	117 (IQI: 90 - 140)
SYNTAX Score*	9 (IQI: 2.5 - 20)
Serum creatinine (mg/L)	1.0 ± 0.7
Ejection fraction < 45%	45 (13.2%)
Killip > I	51 (14%)
Previous CAD	130 (35.6%)
Cardiovascular death	16 (4.4%)

NSTEMI: non-ST-elevation myocardial infarction; STEMI: ST-elevation myocardial infarction; LMC: left main coronary artery;

(*) median (interquartile interval); CAD: coronary artery disease.

### Prognostic value of the SYNTAX Score

**Figure 2:**
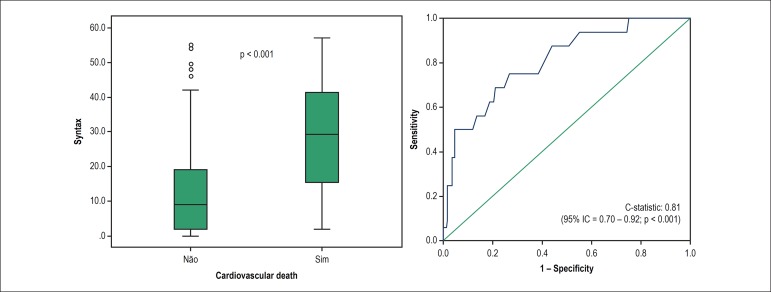
Panel A shows the medians of the SYNTAX Score in individuals who had or did not have in-hospital cardiovascular death (p < 0.001). Panel B represents the C-statistic value of the SYNTAX Score to predict the outcome of cardiovascular death (0.81, 95%CI: 0.70 - 0.92, p < 0.001)

The 16 patients (4.4%) who had in-hospital cardiovascular death had the highest median SYNTAX Score (29, IQI: 14 - 43 *versus* 9, IQI: 2 - 19, p < 0,001). The SYNTAX Score was a significant predictor of cardiovascular death, with C-statistic of 0.81 (95% CI: 0.70 - 0.92; p < 0.001) (Figure 2).

### Independent and incremental prognostic value of the GRACE Score as compared to the SYNTAX Score

**Figure 3:**
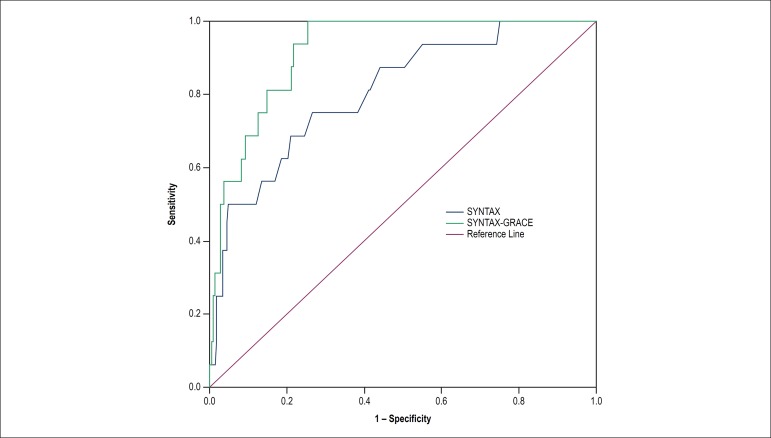
Incremental prognostic value of the SYNTAX-GRACE model as compared to the SYNTAX model to predict the primary outcome

On multivariate logistic regression analysis (Table 2), the GRACE Score was a predictor of in-hospital cardiovascular death after adjusting for the SYNTAX Score (OR = 1.03, 95% CI: 1.01 - 1.04; p < 0.001). The addition of the variable ‘GRACE Score’ to the SYNTAX model caused a significant increment in C-statistic from 0.81 (95% CI: 0.70 - 0.92) to 0.92 (95% CI: 0.87 - 0.96), p = 0.04 (Figure 3). The SYNTAX Score showed proper calibration, with a Hosmer-Lemeshow chi-square test result of 3.53 (p = 0.83). After inclusion of the GRACE Score in the model, the calibration improved, with a chi-square value of 2.73 (p = 0.95).

**Table 2:** Logistic regression model containing the SYNTAX and GRACE Scores to predict the outcome variable.

Variable	Odds Ratio	95%CI	P value
SYNTAX Score (numeric)	1.05	1.01 – 1.09	0.012
GRACE Score (numeric)	1.03	1.01 – 1.04	< 0.01

### Reclassification of the SYNTAX Score by use of the GRACE Score

The IDI analysis showed a mean 9.7% increase in the estimated likelihood of death among the patients who had events, and a 0.45% reduction in the estimated likelihood of death among patients who remained alive. That combination resulted in an IDI of 10.1% (Z score = 2.47; p = 0.01).

## Discussion

This study of a prospective cohort of individuals with ACS assessed the incremental prognostic value of the incorporation of clinical data into an angiographic risk prediction model. There was a clear increment in the prognostic value, represented by a 0.11 gain in C-statistic, when the clinical model (GRACE Score) was incorporated into the SYNTAX Score. Thus, the clinical paradigm provides additional prognostic information for the therapeutic decision-making process after knowledge about the coronary anatomy, and physicians should consider clinical data together with risk stratification by use of coronary angiography.

The prognostic value of the SYNTAX Score in ACS has been assessed in a *post hoc* analysis of the ACUITY Trial, showing higher ischemic event rates for patients scoring in the highest tertiles.^13^ The C-statistic value of the SYNTAX Score found confirms the previous finding, showing a good predictive ability of that score in our population. When assessing the reclassification of the SYNTAX predictive model by the GRACE Score in that population with ACS, the data show that the GRACE Score increments the SYNTAX Score, mainly by detecting candidates for the outcome (sensitivity), without a substantial improvement in the detection of patients who will remain free from the outcome (specificity).

Risk prediction models incorporating clinical and angiographic variables have shown higher predictive accuracy as compared to isolated models in several CAD scenarios.^14-16^ The recently developed SYNTAX Score II consists in the incorporation of clinical data into the original anatomical model, with variables previously tested in a model called Logistic Clinical SYNTAX Score (age, creatinine clearance, and EF),^14^ in addition to the increment of other independent predictors in multivariate analysis, such as the presence of peripheral arterial disease, chronic obstructive pulmonary disease, left main coronary artery lesion and female sex.^17^ Although that model had predictive accuracy and discrimination capacity greater than those of the original anatomical model, it had not been properly tested in the context of patients with ACS. In our study, the increment promoted by the incorporation of clinical data into the original anatomical model was better as compared to that of the Logistic Clinical SYNTAX Score (0.11 vs. 0.09, respectively), suggesting that the incorporation of clinical severity data has greater importance in the ACS scenario.

Our study is one of the few with acute patients, in whom the anatomical complexity is lower, as shown by the median SYNTAX Score of 9 (IQI: 2.5 - 20), similar to that of a previous trial.^13^ Although most patients were considered at low risk by use of the anatomical score, its predictive ability was maintained, and there was a significantly higher incremental value with the incorporation of clinical data as compared to that of previous studies. This might be justified by the fact that the GRACE Score comprises several variables that reflect a higher propensity to complications during the intervention, such as age, heart rate, kidney function and Killip classification. In addition, choosing to use that score in the final model rather than isolated clinical variables allowed for a reduction in the number of patients analyzed, making this analysis more pragmatic, not interfering with its predictive accuracy.

The major limitation of this study is its sample size, with a borderline number of outcomes for the incorporation of the two covariables into the logistic regression analysis. This is related to the generation of hypotheses, which would require randomized clinical trials that incorporated the predictive SYNTAX-GRACE model into the risk stratification process and therapeutic decision-making.

There are significant practical implications in these results. When managing a patient with ACS, the anatomical paradigm usually guides the decision-making process regarding the best revascularization modality. However, we should consider the patient’s predicted clinical risk, even after knowing the coronary anatomy, so that more individualized decision-making processes interfere favorably with the treatment.

## Conclusion

For patients with ACS, clinical data complement the prognostic value of coronary anatomy, and risk stratification should be based on the clinical-anatomical paradigm.
